# Dual Surfactant-Assisted Hydrothermal Engineering of Co_3_V_2_O_8_ Nanostructures for High-Performance Asymmetric Supercapacitors

**DOI:** 10.3390/mi16121334

**Published:** 2025-11-27

**Authors:** Pritam J. Morankar, Aditya A. Patil, Aviraj Teli, Chan-Wook Jeon

**Affiliations:** 1 School of Chemical Engineering, Yeungnam University, 280 Daehak-Ro, Gyeongsan 38541, Republic of Koreaaditya.nanotechnology@gmail.com (A.A.P.); 2 Division of Electronics and Electrical Engineering, Dongguk University-Seoul, Seoul 04620, Republic of Korea

**Keywords:** PVP/SDS, nanoflower morphology, hydrothermal synthesis, pseudocapacitance, asymmetric supercapacitor

## Abstract

This study presents a dual surfactant-assisted hydrothermal approach for the synthesis of Co_3_V_2_O_8_ (CoVO) nanostructures and their surfactant-modified derivatives, PVP-assisted Co_3_V_2_O_8_ (P-CoVO) and PVP–SDS co-assisted Co_3_V_2_O_8_ (P/S-CoVO), which were directly grown on nickel foam. The use of PVP and SDS enabled controlled nucleation and growth, yielding a hierarchical nanoflower-like morphology in P/S-CoVO with increased porosity, a higher surface area, and uniform structural features. Comprehensive physicochemical characterization confirmed that surfactant incorporation effectively modulated particle size, dispersion, and active-site availability. Electrochemical measurements demonstrated that P/S-CoVO exhibited superior performance, with the largest CV area, low equivalent series resistance (0.52 Ω), and a maximum areal capacitance of 13.71 F cm^−2^ at 8 mA cm^−2^, attributable to rapid redox kinetics and efficient ion transport. The electrode also showed excellent cycling stability, retaining approximately 83.7% of its initial capacitance after 12,000 charge–discharge cycles, indicating robust structural integrity and interfacial stability. Additionally, an asymmetric supercapacitor device (P/S-CoVO//AC) delivered a high energy density of 0.082 mWh cm^−2^, a power density of 1.25 mW cm^−2^, and stable operation within a 1.5 V potential window. These results demonstrate that cooperative surfactant engineering provides an effective and scalable strategy to enhance the morphology, electrochemical kinetics, and durability of Co_3_V_2_O_8_-based electrodes for next-generation high-performance supercapacitors.

## 1. Introduction

The twenty-first century is witnessing not only an unprecedented acceleration in global energy demand but also a simultaneous transition toward a low-carbon economy. This transition is complicated by the mismatch between the temporal profiles of renewable energy generation and end-user consumption, where surplus electricity often remains underutilized, while peak demand periods strain supply [1]. Bridging this mismatch requires energy storage technologies that can buffer renewable intermittency with both high energy density and rapid response times [2]. Conventional lithium-ion batteries, though dominant in portable electronics and electric vehicles, are frequently constrained by their kinetic limitations and finite cycle life, while electrostatic capacitors, despite offering ultrafast charge–discharge, provide negligible energy storage [3]. Supercapacitors occupy this intermediate technological niche, delivering high power density and exceptional durability, yet their limited energy density remains a fundamental barrier. Addressing this challenge is less a matter of new device configurations than of innovative electrode materials engineering, where the design of redox-active frameworks with high utilization efficiency is pivotal [4].

Transition-metal oxides (TMOs) are widely recognized as pseudocapacitive electrodes owing to their multiple accessible oxidation states and the ability to host fast and reversible redox reactions at the electrode–electrolyte interface [5]. Representative examples, including RuO_2_, MnO_2_, NiO, Co_3_O_4_, and Fe_2_O_3_, have been investigated extensively, though they are often constrained by poor electronic conductivity and progressive structural degradation under long-term electrochemical operation [6,7]. To mitigate these drawbacks, binary or mixed-metal oxides have attracted growing attention, as the synergistic interactions between two distinct cations frequently enhance electrical conductivity, stabilize the host lattice, and broaden the redox activity [8]. Prominent examples such as NiCo_2_O_4_, MnCo_2_O_4_, ZnCo_2_O_4_, and CuV_2_O_6_ have consistently outperformed their single-metal counterparts [9,10,11]. Within this family, CO_3_V_2_O_8_ nanostructures have emerged as particularly compelling, as they integrate the redox versatility of cobalt (Co^2+/^Co^3+^) with the multivalency of vanadium (V^4+/^V^5+^), thereby furnishing multiple faradaic charge-storage pathways [12]. Moreover, their layered orthorhombic frameworks provide a large number of active redox centers and promote rapid ion transport [13].

The electrochemical promise of CO_3_V_2_O_8_ has been substantiated by several recent investigations. Isacfranklin et al. demonstrated that CO_3_V_2_O_8_ synthesized hydrothermally exhibited superior performance among Ni-, Bi-, and Co-based vanadates, achieving 426.11 F g^−1^ (CV) and 285.65 F g^−1^ (GCD) with 83.64% retention after 5000 cycles [14]. Arasi et al. reported chemically synthesized CO_3_V_2_O_8_ nanostructures with layered morphologies, which delivered a specific capacitance of 790 F g^−1^ at 1 A g^−1^ and preserved 90.1% of their initial capacity after 10,000 cycles [15]. Jiao et al. tailored cobalt vanadate growth on 3D CoO microspheres, yielding hollow nanocages and nanosheets, with optimized electrodes reaching an areal capacitance of 7.58 F cm^−2^ and ASC devices sustaining 84.65% retention over 5000 cycles [16]. Extending beyond CO_3_V_2_O_8_, He et al. synthesized binary CoV_2_O_6_ by a facile co-precipitation route, which exhibited 306.6 F g^−1^ at 1 A g^−1^ and retained 83.3% of its capacitance after 20,000 cycles [17], whereas Sun et al. prepared 3D porous Co_2_V_2_O_7_·3.3H_2_O microflowers with remarkable cycling stability, showing 351 F g^−1^ at 1 A g^−1^ and even 103% retention after 30,000 cycles [18]. More recently, Nguyen et al. fabricated hierarchical CO_3_V_2_O_8_ nanosheet arrays on Ni foam, reporting 878.9 F g^−1^ at 1 A g^−1^ in KOH, which was further enhanced to 1584.5 F g^−1^ with a redox additive; their ASC device achieved 55.5 Wh kg^−1^ at 800 W kg^−1^, underscoring the practical viability of such engineered structures [19]. Collectively, these studies underscore the immense potential of cobalt vanadates, while simultaneously revealing the importance of morphology and surface regulation in unlocking their full electrochemical capability.

Although these studies are promising, pristine CO_3_V_2_O_8_ still suffers from particle agglomeration, limited electronic conductivity, and slow ion transport kinetics, all of which restrict the effective utilization of its redox active sites. Therefore, deliberate structural engineering that controls nucleation, guides crystal growth, and creates well-defined porosity is crucial to overcoming these inherent limitations [20,21,22]. Polyvinylpyrrolidone (PVP), functioning as a nonionic surfactant, coordinates with metal ions via carbonyl groups, acting simultaneously as a stabilizer and growth modulator to suppress aggregation and yield well-dispersed nanostructures [23]. Sodium dodecyl sulfate (SDS), a classical anionic surfactant, complements this effect by providing electrostatic stabilization and micelle-directed porosity, which enhances electrolyte infiltration and accelerates ionic transport [24]. When employed concurrently, PVP and SDS generate a synergistic effect: PVP ensures steric stabilization and controlled particle growth, while SDS introduces hierarchical pore channels and wettability, together producing nanostructured cobalt vanadates with higher surface accessibility, improved conductivity, and superior cycling stability [25]. While several surfactants, such as cetyltrimethylammonium bromide (CTAB), polyethylene glycol (PEG), Triton X-100 (TX-100), hexamethylenetetramine (HMTA), and polyacrylic acid (PAA) have been employed to modulate nanostructure growth in transition-metal oxides, their action is generally limited to either micelle formation or steric stabilization alone. In contrast, the combined use of PVP and SDS offers a complementary and more effective structure-directing environment for cobalt vanadates. PVP coordinates strongly with Co and V ions through its carbonyl group, suppressing uncontrolled nucleation and particle agglomeration, whereas SDS introduces electrostatic stabilization and micelle-guided porosity that enhances electrolyte wettability and ion diffusion. This dual-surfactant strategy enables simultaneous control over crystal growth, dispersion, and hierarchical pore development, which single-surfactant systems cannot achieve. Therefore, PVP and SDS were intentionally selected to produce well-defined, highly accessible Co_3_V_2_O_8_ nanostructures capable of delivering superior pseudocapacitive performance [26,27,28].

While cobalt vanadates have emerged as promising pseudocapacitive materials, their practical performance is fundamentally constrained by particle agglomeration, limited accessible surface area, and slow ion diffusion. Most reported synthesis routes yield Co_3_V_2_O_8_ structures with poorly regulated morphology and restricted porosity, which hinders electrolyte penetration and prevents full utilization of redox-active sites. Moreover, while surfactant-assisted synthesis is recognized as an effective strategy to modulate morphology, systematic insights comparing the individual and synergistic roles of different classes of surfactants in tailoring Co_3_V_2_O_8_ structure and electrochemical behavior remain limited. To address these gaps, the present study introduces a rational dual-surfactant engineering approach using a minimal amount of nonionic PVP and anionic SDS to precisely direct nucleation, regulate crystal growth, prevent aggregation, and introduce hierarchical porosity. This combined strategy enables the formation of a highly open, interconnected cobalt vanadate network with significantly enhanced ion transport and surface accessibility. Compared to the pristine and single-surfactant-modified samples, the optimized P/S-CoVO electrode can delivers markedly superior capacitance, faster charge–discharge kinetics, and improved long-term cycling stability, demonstrating a clear advancement over previous Co_3_V_2_O_8_-based systems. The novelty of this work lies in establishing, for the first time, a comparative and mechanistic understanding of how single vs. dual surfactants cooperatively influence morphology, porosity, and charge-storage efficiency in cobalt vanadate electrodes.

In this work, we present a rational hydrothermal strategy for the synthesis of bare CoVO, P-CoVO, and P/S-CoVO nanostructures, and systematically correlate their structural evolution with electrochemical behavior. By moving stepwise from the pristine oxide to nonionic surfactant-assisted and finally dual surfactant-assisted architectures, this study offers a clear framework to disentangle the role of surfactant chemistry in directing morphology, porosity, and charge-storage kinetics. The optimized P/S-CoVO electrode exhibits markedly superior specific capacitance, rapid charge–discharge capability, and excellent long-term durability compared to the unmodified counterpart. These findings not only validate the effectiveness of dual surfactant-assisted synthesis in unlocking the full electrochemical potential of cobalt vanadates but also establish a broadly applicable design paradigm for engineering hierarchical pseudocapacitive materials for next-generation asymmetric supercapacitors.

## 2. Experimental Section

### 2.1. Materials

Cobalt nitrate hexahydrate (Co(NO_3_)_2_·6H_2_O), ammonium metavanadate (NH_4_VO_3_), PVP(C_6_H_9_NO)_n_, SDS(C_12_H_25_NaSO_4_), and NH_4_OH (25 wt%) were purchased from Sigma-Aldrich (St. Louis, MO, USA) and utilized directly without additional purification. Nickel foam (1 × 2 cm^2^) served as the current collector for electrode preparation. Deionized (DI) water was used for all synthesis and washing steps.

### 2.2. Synthesis of CoVO and Surfactant-Modified Variants

**Figure 1:**
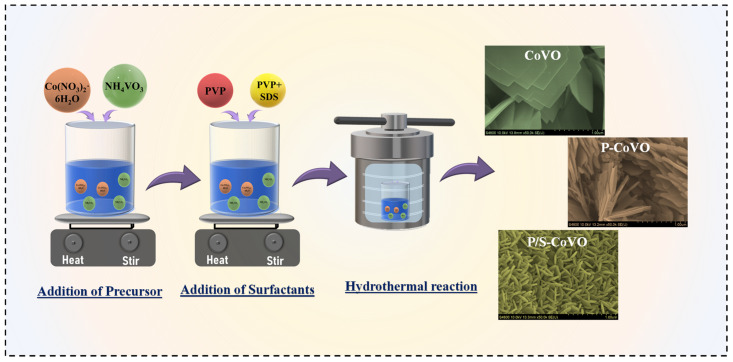
Schematic illustration of the stepwise synthesis of CoVO, P-CoVO, and P/S-CoVO samples through the surfactant-assisted hydrothermal process.

CoVO nanostructures were synthesized on nickel foam using a hydrothermal growth approach. A precursor solution was prepared by dissolving 2 mmol of Co(NO_3_)_2_·6H_2_O and 1.33 mmol of NH_4_VO_3_ in 40 mL of deionized water, followed by pH adjustment to around 9 using NH_4_OH. This pH level ensured steady hydrolysis and the formation of Co–V species suitable for uniform nucleation on the nickel foam, which was immersed directly into the mixture. Surface regulation was achieved by introducing specific surfactants into the precursor. For the P-CoVO sample, 0.003 g of PVP was added before pH adjustment to control nucleation and prevent particle accumulation. In the P/S-CoVO system, 0.003 g of PVP and 0.003 g of SDS were incorporated together to couple steric stabilization with micelle-assisted structuring, promoting a more porous and well-distributed growth on the foam substrate. The assembled solutions were sealed in a 100 mL Teflon-lined autoclave and maintained at 180 °C for 12 h. After the hydrothermal reaction, the coated foams were washed thoroughly with water and ethanol, dried at 60 °C, and finally annealed at 400 °C for 2 h in air to convert the intermediates into crystalline Co_3_V_2_O_8_. The synthesis process of CoVO, P-CoVO, and P/S-CoVO samples is schematically illustrated in Figure 1, showing the stepwise formation and surfactant-assisted structural evolution.

## 3. Sample Characterization and Electrochemical Measurements

The crystal structure, morphological, and surface properties of the synthesized samples (CoVO, P-CoVO, and P/S-CoVO) were investigated using multiple characterization techniques. X-ray diffraction (XRD, PANalytical diffractometer (Birmimgham, UK), Cu Kα radiation, λ = 1.5406 Å) was employed to determine the crystal structure, phase purity, and crystallinity. The surface morphology and microstructural features were examined by field-emission scanning electron microscopy (FE-SEM, S-4800, HITACHI, Tokyo, Japan), while elemental distribution was analyzed by energy-dispersive X-ray spectroscopy (EDS) attached to the microscope. Prior to imaging, the samples were sputter-coated with a thin platinum layer to avoid charging effects. Raman spectroscopy (XploRA Plus, HORIBA Jobin Yvon, Palaiseau, France) was used to probe the vibrational modes and short-range structural ordering, whereas X-ray photoelectron spectroscopy (XPS, K-Alpha, Thermo Scientific, Eastleigh, UK) provided detailed information on surface composition and oxidation states of the elements. Electrochemical properties were assessed using a Biologic WBCS3000 (Gières, France) workstation in a three-electrode configuration, with the CoVO-based electrode as the working electrode, platinum foil as the counter electrode, and Ag/AgCl as the reference electrode, in 2 M KOH aqueous electrolyte. Cyclic voltammetry (CV), galvanostatic charge–discharge (GCD), and electrochemical impedance spectroscopy (EIS, 0.01 Hz–100 kHz) were employed to evaluate specific capacitance, rate performance, and charge-transfer resistance. Cycling durability was examined by extended GCD measurements. For asymmetric supercapacitor (ASC) fabrication, the optimized P/S-CoVO electrode served as the positive electrode and activated carbon (AC) as the negative electrode, with device performance evaluated in terms of capacitance, energy density, power density, and stability.

## 4. Results and Discussion

### 4.1. X-Ray Diffraction Elucidation

**Figure 2:**
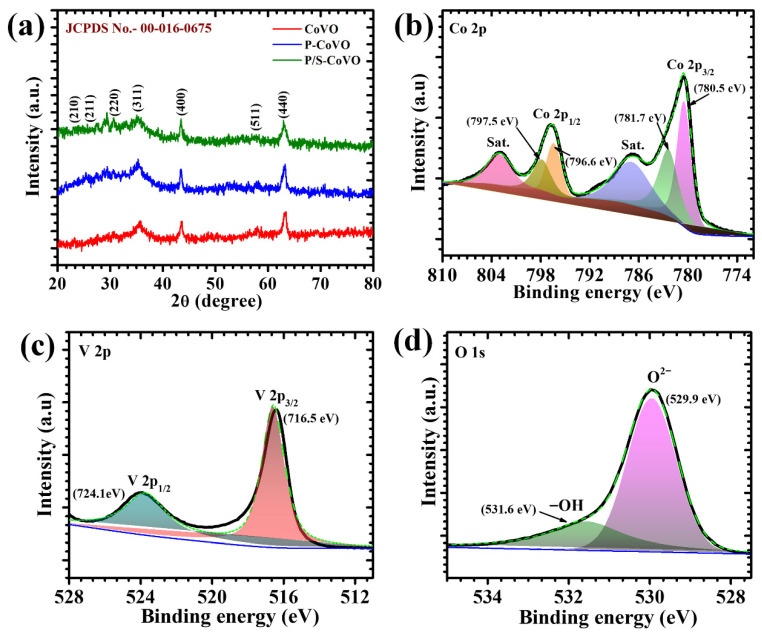
(**a**) XRD patterns of pristine CoVO, P-CoVO, and P/S-CoVO samples, along with the high-resolution spectra of (**b**) Co 2p, (**c**) V 2p and (**d**) O 1s for the P/S-CoVO sample.

The structural features of the synthesized materials were analyzed using XRD, and the corresponding diffraction patterns are presented in Figure 2a. All the diffraction peaks recorded for CoVO, P-CoVO, and P/S-CoVO are highly consistent with the standard JCPDS card No. 00-016-0675, affirming the successful formation of phase-pure cubic Co_3_V_2_O_8_ [29]. No secondary reflections attributable to cobalt oxides or vanadium oxides were detected, indicating that the hydrothermal process followed by annealing yielded single-phase materials in all cases [30]. The major reflections observed at 2θ ≈ 23.9°, 26.2°, 30.3°, 35.7°, 43.5°, 57.5° and 63.2° can be indexed to the (210), (211), (220), (311), (400), (511), and (440) planes of cubic Co_3_V_2_O_8_.The sharp and well-defined peaks across all samples suggest good crystallinity and structural stability. It is noteworthy that the diffraction profiles of P-CoVO and P/S-CoVO remain identical to that of pristine CoVO, demonstrating that the incorporation of surfactants did not modify the intrinsic crystal phase of Co_3_V_2_O_8_ [31]. This observation is significant because it shows that while PVP and SDS influence particle growth, dispersion, and the development of porous architectures, the underlying crystal lattice remains preserved. This behavior can be attributed to the distinct roles of PVP and SDS during hydrothermal synthesis. PVP is a nonionic surfactant whose carbonyl (C=O) groups coordinate with metal cations, providing steric stabilization and regulating nucleation by forming surface-bound complexes that suppress uncontrolled agglomeration and promote uniform particle evolution [32]. While, SDS, an anionic surfactant, contributes electrostatic stabilization and micelle-directed self-assembly; its negatively charged sulfate head groups adsorb on particle surfaces, promoting the formation of porous and well-dispersed architectures while preventing lattice distortion [33]. Because both PVP and SDS primarily modulate growth kinetics and surface energy rather than participating in lattice substitution, their incorporation does not disrupt the intrinsic framework of Co_3_V_2_O_8_, thereby preserving the original phase while markedly improving morphological uniformity and surface accessibility. Such structural consistency is crucial for electrochemical studies, as phase purity and lattice stability directly ensure reliable redox activity and reproducible charge–storage behavior. The XRD analysis thus confirms that all three synthesized samples retain the cubic Co_3_V_2_O_8_ framework, establishing a robust structural foundation upon which surfactant-assisted morphological tuning can be correlated with enhanced supercapacitor performance [17,34].

### 4.2. X-Ray Photoelectron Spectroscopy

The surface chemistry and corresponding oxidation states of the optimized P/S–CoVO electrode were examined through high-resolution XPS, as presented in Figure 2b–d. The Co 2p spectrum (Figure 2b) shows two principal peaks at 780.5 eV and 796.6 eV, consistent to Co 2p_3/2_ and Co 2p_1/2_, respectively. Additional satellite peaks at 781.7 eV and 797.5 eV confirm the coexistence of Co^2+^ and Co^3+^ species, revealing a mixed-valence cobalt environment [35]. Such a configuration is advantageous for promoting reversible redox reactions, thereby contributing to enhanced pseudocapacitive charge storage [36]. The V 2p spectrum (Figure 2c) demonstrates characteristic peaks at 716.5 eV and 724.1 eV, allotted to V 2p_3/2_ and V 2p_1/2_, respectively. The presence of both V^4+^ and V^5+^ oxidation states suggest multiple redox-active centers that facilitate electron transfer and improve reaction kinetics. The O 1s spectrum (Figure 2d) shows three distinct features, with the main peak at 529.9 eV assigned to lattice oxygen, while the other two peaks at 531.6 eV and 532.4 eV are related to surface hydroxyl groups and adsorbed oxygen species or oxygen vacancies, respectively. These oxygen-related defects are known to enhance electrolyte accessibility and accelerate interfacial charge transport at the electrode–electrolyte interface. The XPS analysis clearly demonstrates that the dual-surfactant strategy modulates the surface chemistry of CoVO, leading to a balanced distribution of mixed-valence metal species and oxygen-related defects [37]. Such a surface configuration enhances the electronic environment and fosters rapid redox exchanges, directly translating into improved charge storage capability and stability. The findings emphasize that controlled surface tailoring through surfactant mediation is a decisive factor in achieving efficient electrochemical performance in CoVO-based electrodes [38].

### 4.3. Morphological and Elemental Composition

**Figure 3:**
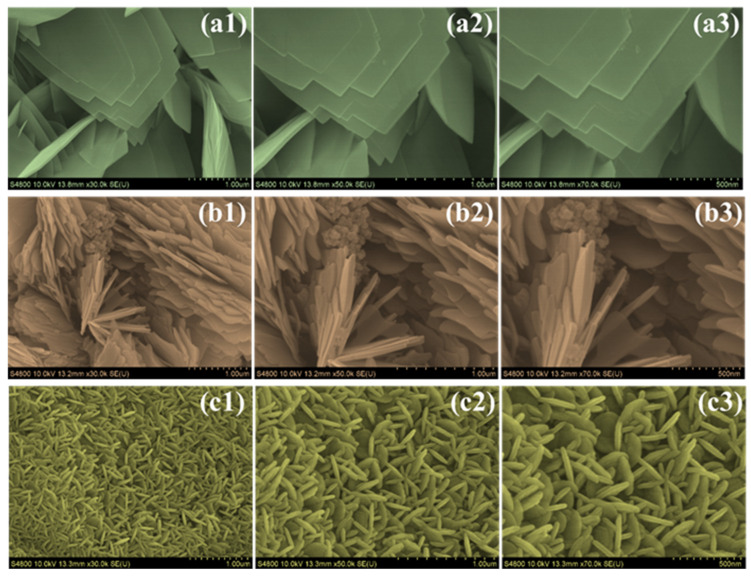
FESEM images of (**a1**–**a3**) CoVO, (**b1**–**b3**) P-CoVO, and (**c1**–**c3**) P/S-CoVO samples captured at different magnifications.

FESEM analysis (Figure 3) clearly reveals a distinct and progressive morphological evolution across the CoVO, P-CoVO, and P/S-CoVO samples, demonstrating the significant influence of surfactant chemistry on crystal growth and structural organization. The CoVO sample, as shown in Figure 3(a1–a3), displays large, densely packed plate-like crystallites arranged in thick stacked domains with very limited interparticle spacing [39]. This compact structure restricts electrolyte mobility and provides fewer exposed edge sites for Faradaic reactions, resulting in a relatively less active surface. When PVP is introduced, the P-CoVO sample, illustrated in Figure 3(b1–b3), exhibits a noticeable change toward a more open and texturally diverse morphology. The stacked plates become thinner and partially separated, and several regions develop radially protruding blade-like features. These structural changes arise from the steric effect of PVP during synthesis, which controls excessive lateral aggregation, improves mesoporosity, and enhances electrolyte accessibility compared to the pristine sample [40]. A significant morphological transformation is observed in the P/S-CoVO sample, as shown in Figure 3(c1–c3), where the combined influence of PVP and SDS directs the formation of a hierarchical nanoflower-like structure composed of interconnected nanoplates. This three-dimensional framework is highly porous and interconnected, which greatly enhances ion diffusion and electron transport. The cooperative interaction between the electrostatic soft-templating action of SDS and the steric stabilization of PVP promotes anisotropic growth, resulting in a well-organized nanoflower morphology instead of the compact plate-like texture observed in the other samples. This hierarchical and open architecture offers abundant electroactive sites, facilitates rapid electrolyte infiltration, and maintains strong structural stability during repeated charge–discharge cycles. As a result, the P/S-CoVO sample exhibits the most favorable morphology, providing an optimized framework that enables smooth ion transport, faster charge-transfer kinetics, and excellent electrochemical performance in asymmetric supercapacitor devices [41].

Additionally, FESEM analysis of the SDS-assisted CoVO (SDS–CoVO) sample, shown in Figure S1(a1–a3), reveals the formation of densely packed, elongated rod-like nanostructures with smooth surfaces and uniform thickness. These nanorods exhibit lateral dimensions in the range of several hundred nanometers and are interconnected to form a highly entangled 3D network. The presence of SDS, an anionic surfactant, likely promotes anisotropic growth by selectively adsorbing onto specific crystallographic facets of CoVO nuclei, thereby directing the formation of elongated 1D structures. This micelle-assisted growth mechanism enhances dispersion and suppresses aggregation, resulting in a more ordered and rod-dominated morphology compared to the irregular, plate-like structures observed in pristine CoVO. Although SDS-CoVO is not part of the core comparison set of this study, its morphological evaluation provides valuable insight into the individual influence of SDS on crystal growth and supports the rationale for employing dual-surfactant engineering in the optimized P/S-CoVO electrode.

**Figure 4:**
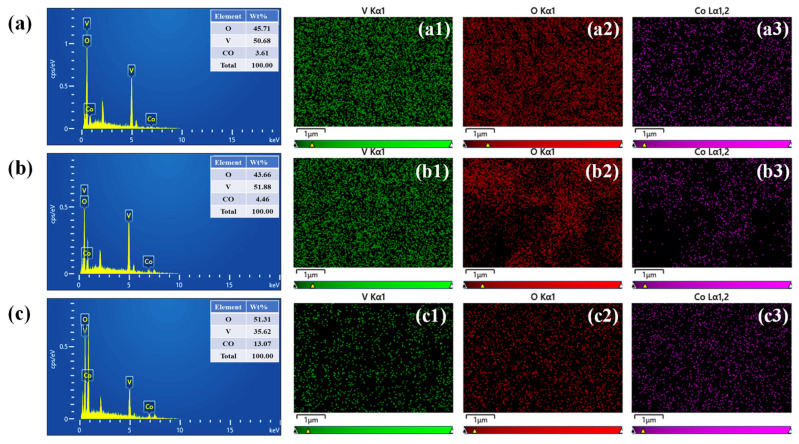
(**a**–**c**) EDS spectra and (**a1**–**c3**) elemental mapping of CoVO, P-CoVO, and P/S-CoVO samples.

In this work, EDS and elemental mapping analyses were performed to verify the elemental composition and spatial distribution of Co, V, and O in the synthesized CoVO, P-CoVO, and P/S-CoVO samples. The corresponding EDS spectra shown in Figure 4a–c provide both qualitative and quantitative information about the elemental constituents. The spectra confirm the exclusive presence of Co, V, and O, indicating that all samples possess high chemical purity without any detectable impurities. The compositional data presented in the accompanying table further support the successful synthesis and compositional stability of all samples. Elemental color mapping was also employed to assess the uniformity of element dispersion across the surface of each sample. In the pristine CoVO sample (Figure 4(a1–a3)), Co, V, and O are uniformly distributed, confirming the homogeneity of the material. The P-CoVO sample shown in Figure 4(b1–b3) exhibits a similar uniform distribution pattern, suggesting that the incorporation of PVP improves morphological uniformity without affecting the elemental composition. Meanwhile, the P/S-CoVO sample (Figure 4(c1–c3)) shows an even and well-dispersed distribution of Co, V, and O across the hierarchical nanoflower-like framework. This consistent and homogeneous elemental distribution aligns with the observed refined morphology, highlighting the synergistic effect of PVP and SDS in promoting uniform crystal growth and preserving excellent compositional integrity throughout the structure.

## 5. Electrochemical Analysis

**Figure 5:**
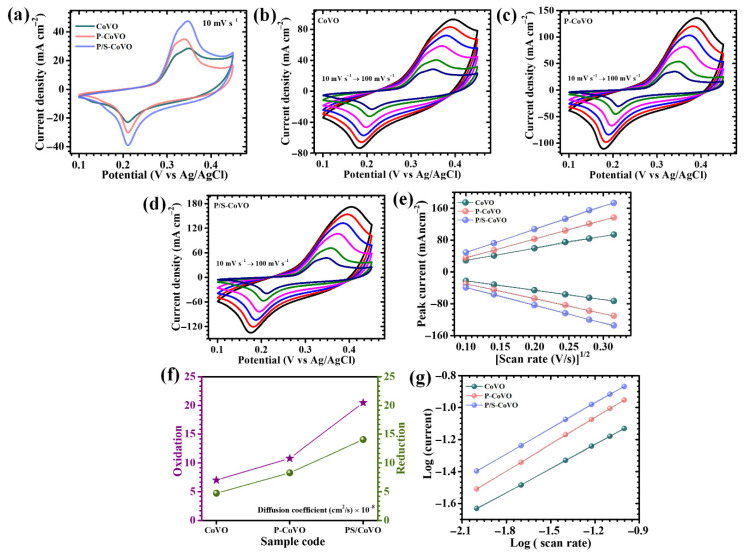
Cyclic voltammetry of (**a**) all CoVO electrodes at a scan rate of 10 mV s^−1^ within a potential window of 0.1 to 0.45 V, CV curves of (**b**) CoVO, (**c**) P-CoVO, and (**d**) P/S-CoVO samples at different scan rates (10–100 mV s^−1^), (**e**) plot of peak current versus the square root of scan rate, (**f**) graphical representation of the calculated diffusion coefficient, and (**g**) plot of log(i) versus log(scan rate).

The electrochemical characteristics of the Co_3_V_2_O_8_ electrodes synthesized under different surfactant environments, were systematically evaluated to understand the influence of single and dual-surfactant-assisted morphology control on their capacitive behavior.CV, GCD, and EIS analyses were performed in a conventional three-electrode setup employing 2 M KOH as the aqueous electrolyte. Figure 5a presents the CV responses of all electrodes measured within a potential range of 0.1–0.45 V at a scan rate of 10 mV s^−1^. All three electrodes exhibit distinct and well-defined anodic and cathodic peaks, confirming the pseudocapacitive nature of the charge storage process, which arises from reversible faradaic reactions involving cobalt and vanadium ions. These redox features are associated with the reversible transitions between Co^2+^/Co^3+^ and V^5+^/V^4+^ oxidation states, reflecting efficient electron-proton-coupled transfer reactions during the charge–discharge process [20]. The overall pseudocapacitive mechanism governing Co_3_V_2_O_8_-based electrodes can be represented by the following redox reaction (1) [20]:

(1) $${Co}_{3}V_{2}O_{8}+H_{2}O+{OH}^{-}⇌CoOOH+VO{\left(OH\right)}_{2}+e^{-}$$

This equation highlights the cooperative contribution of both cobalt and vanadium centers toward the faradaic energy storage mechanism. The electrochemical activity of Co_3_V_2_O_8_ is largely determined by the availability of these redox-active sites and the efficiency of ion transport within the electrode-electrolyte interface. In Co_3_V_2_O_8_, charge storage primarily relies on the intrinsic Co^2+^/Co^3+^ and V^5+^/V^4+^ transitions; however, its performance is often limited by sluggish ionic mobility and the agglomeration of particles, which diminish the effective surface area available for electrochemical reactions [42]. The incorporation of surfactants during synthesis plays a crucial role in mitigating these limitations. The addition of PVP or the hybrid combination of PVP and SDS modifies the nucleation and growth pathways, resulting in tailored morphologies and enhanced surface architectures. To further examine the rate-dependent electrochemical response, CV measurements were conducted over a range of scan rates (10–100 mV s^−1^) for all three electrodes, as displayed in Figure 5b–d. A consistent rise in current density was observed with higher scan rates for each electrode, indicating excellent reversibility and mechanical robustness during rapid charge–discharge cycles. The preservation of CV shape across varying scan rates reflects stable redox kinetics and minimal structural distortion under high-rate conditions. Furthermore, the near-symmetric nature and reproducible profiles of the CV curves suggest favorable charge transfer dynamics and efficient ion diffusion throughout the electrode matrix. Among the investigated samples, the P/S-CoVO electrode exhibits the highest redox peak intensity and the largest integrated CV area, signifying its superior charge storage capability compared to CoVO and P-CoVO. This outstanding electrochemical behavior is due to the synergistic interaction between PVP and SDS during synthesis, which promotes homogeneous nucleation, controlled particle assembly, and the evolution of a uniform, porous, and hierarchically interconnected nanoflower-like structure. Such an architecture facilitates fast ion transport, improved electrolyte penetration, and the effective utilization of electroactive sites, thereby maximizing pseudocapacitive behavior [43]. In contrast, the CoVO electrode synthesized without any surfactant exhibits a relatively dense and think microstructure with limited porosity, restricting ion accessibility and reducing the number of available redox centers. Similarly, the P-CoVO sample, though improved in comparison to CoVO, still demonstrates partially aggregated and compact features that hinder efficient electrolyte diffusion and slow the kinetics of redox transitions. Consequently, the hybrid surfactant-assisted P/S-CoVO electrode achieves a superior combination of structural openness, electronic connectivity, and electrochemical activity, establishing it as the most optimized composition among the series.

To better understanding insight into the redox kinetics and ion diffusion behavior of the synthesized electrodes, the correlation between the peak current density (*i_p_*) and the square root of the scan rate (*v*^1/2^) was carefully analyzed. As illustrated in Figure 5e, all Co_3_V_2_O_8_-based electrodes exhibit a strong linear relationship between *i_p_* and *v*^1/2^, signifying that the charge storage mechanism is mainly regulated by a diffusion-driven process rather than surface-limited reactions. This linear dependence indicates that ionic migration within the electrode-electrolyte interface has a major impact on the overall electrochemical performance. To quantitatively evaluate ion diffusion, the apparent diffusion coefficients (D) were calculated using the classical Randles–Sevcik equation, expressed as (2) [5]:

(2) $$D=\frac{i_{p}}{2.69\times 10^{5}\times n^{3/2}\times A\times C\times v^{1/2}}$$

where *i_p_* represents the redox peak current density, *v*^1/2^ is the square root of the scan rate, *n* is the number of electrons transferred in the redox process, *A* denotes the electrochemically active surface area, and *C* is the electrolyte concentration. The diffusion coefficients estimated at a scan rate of 10 mV s^−1^ are presented in Table 1 and explicitly depicted in Figure 5f for comparative assessment. Amongst the investigated samples, the hybrid P/S-CoVO electrode exhibits the highest diffusion coefficient values for both anodic and cathodic reactions, revealing its superior ion mobility and efficient charge transfer capability. This significant enhancement can be ascribed to the cooperative interaction between PVP and SDS during the synthesis process, which induces significant modifications in surface chemistry and structural architecture. The combined presence of these additives creates a highly porous and interconnected framework that enhances electrolyte infiltration and accelerates redox kinetics by shortening the ion diffusion pathways. Conversely, the pristine CoVO and single-polymer-assisted P-CoVO electrodes display comparatively lower diffusion coefficients, indicative of hindered ion transport and slower electrochemical response. Although the incorporation of PVP alone improves structural uniformity to some extent, it does not fully eliminate diffusion resistance.

To gain comprehensive insight into the underlying charge storage mechanism of all electrodes, Dunn’s analytical approach was employed. This well-established method effectively distinguishes between capacitive (surface-controlled) and diffusion-controlled (bulk ion insertion) processes by examining the relationship between the peak current (*i_p_*) and the scan rate (*ν*). The dependence follows a power-law behavior, which can be mathematically expressed as (3) and (4) [44,45]:

(3) $$i\;={av}^{b}$$

(4) $$\log\left(i\right)=\log\left(a\right)+b\;log(v)$$

Here, *a* and *b* are adjustable constants, and the slope b derived from the *log*(*i*) plot vs. the *log*(*v*) plot (Figure 5g) provides key mechanistic insight into the charge storage behavior. A b-value close to 1 suggests that the process is dominated by surface-controlled capacitive reactions, whereas a value near 0.5 implies that the kinetics are primarily governed by diffusion-limited faradaic processes involving ion intercalation within the electrode matrix. In the present investigation, the calculated b-values for the CoVO, P-CoVO, and P/S-CoVO electrodes ranged from 0.51 to 0.56 (Table 1), signifying that their charge storage mechanisms are largely diffusion-controlled. This indicates that OH^−^ ions from the electrolyte actively participate in reversible intercalation and deintercalation within the Co_3_V_2_O_8_ lattice, contributing to faradaic energy storage rather than simple electrostatic adsorption.

To further decouple and quantify the relative contributions from capacitive and diffusion-controlled processes, the total current response was analyzed using the following kinetic model (5) [46]:

(5) $$i\left(V\right)=k_{1}v{\;+\;k_{2}v}^{1/2}$$

**Figure 6:**
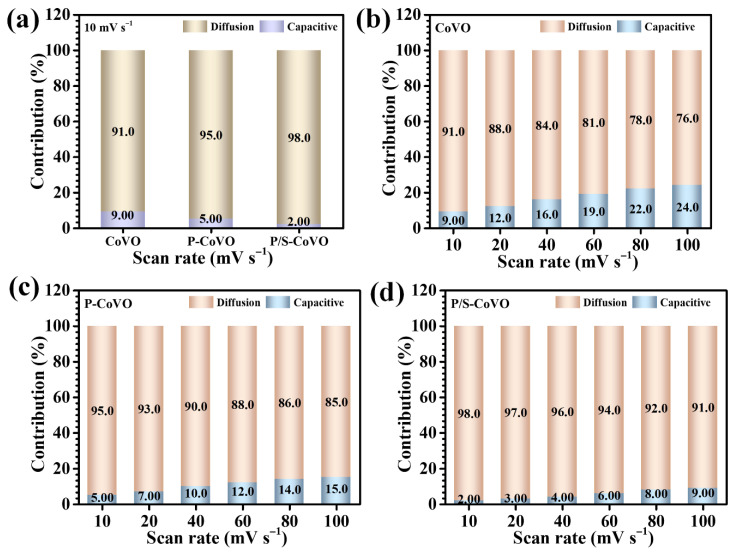
Capacitive and diffusion-controlled contributions of (**a**) all CoVO electrodes at a scan rate of 10 mV s^−1^, and at different scan rates for (**b**) CoVO, (**c**) P-CoVO, and (**d**) P/S-CoVO samples.

In this equation, *k*_1_*ν* represents the capacitive (surface-related) contribution, whereas *k*_2_*ν*^1/2^ corresponds to the diffusion-governed (bulk-controlled) component. By plotting *i*(*V*)/*ν*^1/2^ vs. *ν*^1/2^, the constants *k*_1_ and *k*_2_ were extracted to determine the respective contributions. At a representative scan rate of 10 mV s^−1^ (Figure 6a), the diffusion-controlled fraction was estimated to be approximately 91% for CoVO, 95% for P-CoVO, and 98% for P/S-CoVO electrodes. These results clearly highlight that the hybrid polymer-assisted P/S-CoVO electrode exhibits the highest proportion of diffusion-dominated charge storage, underscoring its superior ability to facilitate ion transport and redox kinetics. This enhancement is attributed to its highly porous, flower-like hierarchical morphology, engineered through the synergistic interplay of PVP and SDS, which provides extensive active surface area, abundant diffusion channels, and efficient electrolyte accessibility. In contrast, the pristine CoVO and singly modified P-CoVO samples show relatively moderate diffusion participation due to their compact, aggregated surface structures that impede ionic movement and restrict access to internal redox sites. Although PVP assistance alone partially mitigates particle clustering, the addition of SDS in the hybrid system significantly improves structural openness and connectivity, enabling simultaneous electron conduction and ion diffusion [47]. The effect of varying scan rate further validates these observations (Figure 6b–d). At higher scan rates, all electrodes demonstrated an increased capacitive contribution, as rapid potential sweeps hinder deep ion penetration into the lattice, leading to surface-dominated charge accumulation. Conversely, at slower scan rates, diffusion-controlled redox processes become more prominent, consistent with the b-values approaching 0.5 [48,49]. Notably, the P/S-CoVO electrode maintains a well-balanced response between surface and diffusion contributions across all scan rates, reflecting its optimized architecture and enhanced kinetics. The dual-surfactant strategy effectively tailors the external morphology and internal porosity of Co_3_V_2_O_8_, thereby achieving a desirable equilibrium between energy-oriented diffusion processes and power-oriented capacitive behavior, key to its outstanding electrochemical performance.

**Figure 7:**
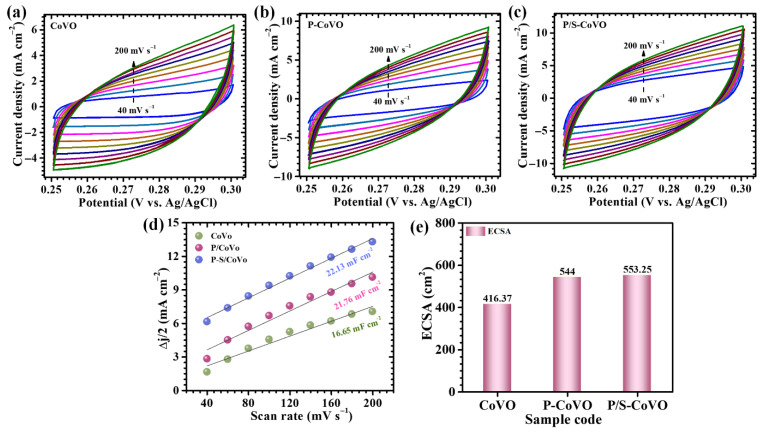
(**a**–**c**) CV recorded for the three electrodes at various scan rates confined to the non-faradaic region, (**d**) corresponding linear fit of current response versus scan rate for determining the double-layer capacitance (C_dl_), and (**e**) calculated ECSA values for the fabricated electrodes.

To evaluate the electrochemically active surface area (ECSA) of the CoVO, P-CoVO, and P/S-CoVO electrodes, CV was performed at different scan rates within a carefully selected non-faradaic potential window (Figure 7a–c). Limiting the measurements to this region ensures that the recorded current arises purely from double-layer capacitance, without interference from faradaic redox processes. The capacitive currents obtained at various scan rates were used to extract the double-layer capacitance (C_dl_), as shown in Figure 7d. The ECSA was then calculated using Equation (6) [5],

(6) $$ECSA=\frac{c_{dl}}{C_{s}}$$

where *C_dl_* represents the derived double-layer capacitance and *C_s_* is the specific capacitance of a surface in 1 M KOH (0.04 mF cm^−2^). This method provides a reliable estimation of the accessible electroactive surface area participating in charge storage. Based on this analysis, the CoVO, P-CoVO, and P/S-CoVO electrodes exhibit ECSA values of approximately 416.37, 544, and 553.25 cm^2^, respectively (Figure 7e). The markedly increased ECSA of the P/S-CoVO electrode reflects its highly porous, interconnected architecture, offering abundant accessible active sites and rapid ion transport pathways. This enlarged electroactive surface area directly correlates with its superior capacitive performance.

**Figure 8:**
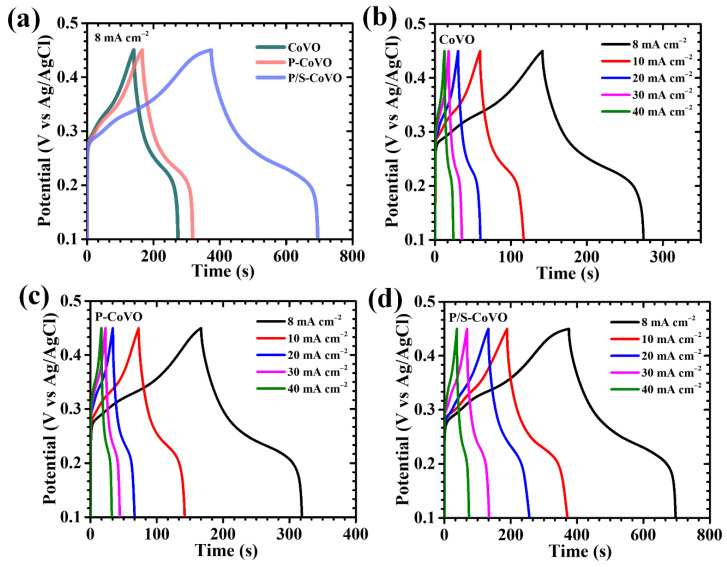
GCD curves of (**a**) all CoVO electrodes at a current density of 8 mA cm^−2^, and GCD plots of (**b**) CoVO, (**c**) P-CoVO, and (**d**) P/S-CoVO samples at various current densities.

**Figure 9:**
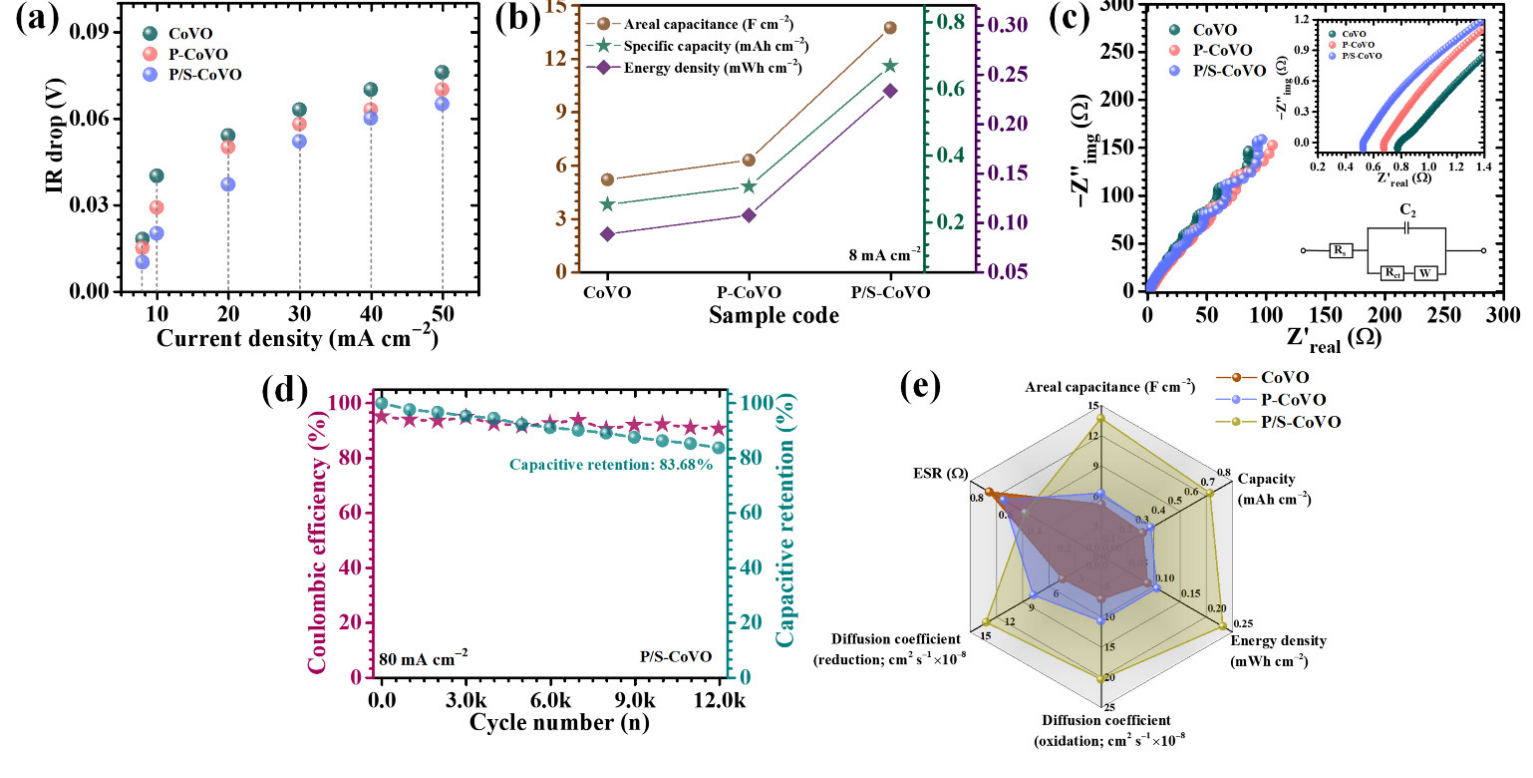
(**a**) IR-drop analysis showing internal resistance, (**b**) comparison of areal capacitance, specific capacity, and energy density of CoVO electrodes, (**c**) Nyquist plot of CoVO electrodes, (**d**) cyclic stability over 12,000 GCD cycles for the P/S-CoVO sample, and (**e**) radar chart summarizing the key electrochemical parameters of the P/S-CoVO electrode.

Figure 8a illustrates the GCD profiles of the CoVO, P-CoVO, and P/S-CoVO electrodes recorded at a current density of 8 mA cm^−2^ within a potential window of 0.1 to 0.45 V. All electrodes exhibit distinct nonlinear charge–discharge curves with visible voltage plateaus, characteristic of diffusion-controlled faradaic reactions typically observed in battery-type pseudocapacitive systems [50]. Notably, the P/S-CoVO electrode displays a more pronounced curvature and a smoother discharge path, indicative of efficient and reversible OH^−^ ion intercalation along with rapid redox transitions at the electrode-electrolyte interface [50]. The markedly extended discharge duration of the P/S-CoVO electrode compared with CoVO and P-CoVO confirms its superior charge-storage capacity, which directly stems from the optimized structural features introduced through the dual-surfactant-assisted synthesis. The current-density-dependent GCD measurements (8–40 mA cm^−2^, Figure 8b–d) further corroborate these findings. Across all applied currents, the electrodes preserve their characteristic voltage plateaus consistent with the redox couples identified in CV analysis, reaffirming the dominance of pseudocapacitive processes. Nearly symmetrical charge and discharge curves were observed for all samples, demonstrating high coulombic efficiency, minimal polarization effects, and stable redox reversibility. Among them, the P/S-CoVO electrode exhibited the most ideal symmetry and an extremely low IR drop at the onset of discharge, reflecting improved electron conductivity and accelerated ion transport kinetics. The near-linear potential response during the charge–discharge sequence confirms the structural integrity of the electrode and its ability to sustain stable redox cycles under repeated operation. The IR-drop analysis presented in Figure 9a provides further insight into the charge-transport dynamics. For all electrodes, a gradual decrease in IR drop was observed as the current density decreased, indicating reduced resistive losses at slower charge–discharge rates. Remarkably, the P/S-CoVO electrode consistently recorded the lowest IR-drop values across the entire range, highlighting its minimized internal resistance and enhanced interfacial charge transfer.

To quantitatively assess electrochemical performance, areal capacitance (C_A_), energy density (ED), and power density (PD) were calculated using the following expressions, which accurately account for the nonlinear GCD profiles typical of faradaic pseudocapacitance: (7)–(9) [6,51]:

(7) $$C_{A}=\frac{I\times 2\times \int V\left(t\right)dt}{A\times {\left(∆V\right)}^{2}}$$

(8) $$ED=\frac{1}{2\times 3600}\;C_{A}\times {dV}^{2}$$

(9) $$PD=\frac{ED\times 3600}{T_{d}}$$

where *I* represents the applied current, *∫V*(*t*)*dt* is the integrated discharge area, *A* denotes the geometric electrode area (cm^2^), *ΔV* is the potential window, and *T_d_* is the discharge time. These equations offer a more precise evaluation of electrochemical performance by integrating the intrinsic nonlinearity of pseudocapacitive behavior. At a current density of 8 mA cm^−2^, the calculated areal capacitances for the CoVO, P-CoVO, and P/S-CoVO electrodes were found to be 5.172, 6.269, and 13.714 F cm^−2^, respectively (Figure 9b and Table 2). The exceptionally high capacitance achieved by the hybrid P/S-CoVO sample clearly demonstrates the effectiveness of combining PVP and SDS during synthesis. This improvement arises from several synergistic factors, including the formation of a highly porous, flower-like network that provides a vast density of electroactive sites, enhances electrolyte infiltration, and shortens ion-diffusion paths through its open interconnected channels. The variation in capacitance and energy density with increasing current density (Table 2) reveals a systematic decline across all electrodes, a typical trend associated with kinetic limitations of OH^−^ ion transport at high charge–discharge rates. At elevated currents, the diffusion of ions into deeper lattice sites becomes restricted, and surface redox reactions dominate the charge storage process [52]. Despite this expected reduction, the P/S-CoVO electrode retained 38.1% of its initial capacitance at 40 mA cm^−2^, highlighting its excellent rate capability and mechanical stability under high-rate conditions. Overall, the comprehensive GCD analysis confirms that the hybrid P/S-CoVO electrode offers a highly optimized electrochemical architecture characterized by superior conductivity, reduced polarization, extensive surface accessibility, and rapid ion-electron transport. The rational design enabled by the dual-surfactant strategy effectively integrates both PVP’s stabilizing function and SDS’s dispersing and pore-forming role, thereby producing a structurally robust electrode capable of delivering outstanding charge-storage capacity and high-rate performance compared with the pristine and singly modified counterparts.

EIS measurements were carried out at 10 mV amplitude over a frequency range of 10 kHz–0.01 Hz to assess the charge-transport and interfacial behavior of Co_3_V_2_O_8_ electrodes. The Nyquist plots (Figure 9c), displaying the imaginary (−Z″) versus real (Z′) components, were interpreted using solution resistance (ESR), charge-transfer resistance, and a Warburg diffusion element [53]. The EIS spectra of CoVO, P-CoVO, and P/S-CoVO electrodes were fitted using a modified Randle circuit (R1 + C2/R2 + W3, inset of Figure 8c), where R1 is the series resistance, R2 is the charge-transfer resistance, C2 represents the double-layer capacitance, and W3 is the Warburg element accounting for ion diffusion. The fitted parameters (Table 1) reveal that the P/S-CoVO electrode exhibits the lowest R1 (0.52 Ω) and R2 (2.134 Ω), indicating enhanced electrical conductivity, faster interfacial charge transfer, and improved ion diffusion compared to CoVO and P-CoVO. At lower frequencies, the P/S-CoVO electrode displayed a more vertical line compared to CoVO and P-CoVO, confirming enhanced capacitive behavior and rapid ion diffusion. The improved EIS response is attributed to the synergistic role of PVP and SDS, which induce a porous, interconnected nanostructure that promotes ion/electron transport and reduces resistive losses. The progressive decrease in ESR across CoVO to P-CoVO to P/S-CoVO is a direct consequence of surfactant-mediated improvements in particle-particle contact, and electrolyte accessibility. Pristine CoVO suffers from dense thick domains and poor interconnectivity between adjacent plates, which increases its ohmic resistance and limits fast electron movement across the electrode. Upon introducing PVP, it suppressing uncontrolled growth and significantly reducing particle clustering. This results in more uniformly distributed features with enhanced electrical contact between grains, thereby lowering the intrinsic electronic resistance (R1). When SDS is additionally incorporated, its anionic head groups and micelle-forming behavior generate a more open, microporous/hierarchical morphology. This structural rearrangement markedly improves electrolyte infiltration, reduces the ion-diffusion path length, and minimizes interfacial charge-transfer resistance (R2). The increased surface wettability imparted by SDS also promotes more complete electrolyte contact with electroactive sites, further lowering interfacial resistance. The combined steric stabilization of PVP and electrostatic/micellar templating of SDS yield a highly interconnected, conductive, and electrolyte-permeable network. Such structural and interfacial optimization directly correlates with the improved electrochemical kinetics observed in EIS and GCD analyses [54,55].

Long-term stability under high current densities is a key standard for evaluating the real-world practical potential of supercapacitor electrodes. To evaluate this, the long-term electrochemical stability of the P/S-CoVO electrode was investigated through constant GCD testing conducted at 80 mA cm^−2^ for 12,000 cycles. The evolution of capacitance retention and coulombic efficiency as a function of cycle number is illustrated in Figure 9d. During the initial stage of cycling, the electrode exhibited a slight rise in capacitance, which is attributed to the progressive electrochemical activation process. This activation involves gradual wetting of the electrode surface, improved electrolyte infiltration into inner porous regions, and enhanced accessibility of previously inactive redox sites. After stabilization, the P/S-CoVO electrode maintained a highly consistent performance, showing negligible decay in capacitance even after prolonged cycling. Remarkably, after 12,000 cycles, it retained 83.68% of its initial capacitance, corresponding to only ~16% loss, demonstrating superior cycling endurance and robust electrochemical integrity. This outstanding stability reflects the mechanical resilience and chemical durability of the hybrid electrode framework. The synergistic incorporation of PVP and SDS produces a flexible, interconnected network capable of accommodating strain from repeated ion intercalation/deintercalation processes. Such surface morphology effectively mitigates internal stress, prevents particle cracking or delamination, and preserves the structural coherence of the redox-active surface. Furthermore, the coulombic efficiency remained above 90% throughout the entire test, indicating excellent reversibility of the redox reactions and minimal parasitic side reactions or resistive polarization. Overall, the combined evidence of exceptional capacitance retention, structural robustness, and stable coulombic efficiency confirms that the P/S-CoVO electrode can sustain rapid and reversible faradaic processes under long-term cycling.

The radar chart in Figure 9e offers a comprehensive assessment of key electrochemical parameters, areal capacitance, energy density, diffusion coefficient, and ESR, for CoVO, P-CoVO, and P/S-CoVO electrodes. The P/S-CoVO electrode exhibits the most expanded and balanced profile, signifying its superior electrochemical performance. Its high capacitance, large diffusion coefficient, and low ESR collectively confirm efficient ion/electron transport and abundant active site accessibility. In contrast, P-CoVO shows moderate improvement, while pristine CoVO displays the weakest performance due to limited porosity and higher internal resistance. The remarkable enhancement in P/S-CoVO arises from the synergistic role of PVP and SDS, which co-regulate nucleation and pore formation, yielding a uniform, hierarchical nanoflower network. This optimized architecture ensures balanced kinetics, superior charge storage, and exceptional rate performance, validating the effectiveness of the dual-surfactant engineering approach.

To contextualize the superior performance of our P/S-CoVO electrode, Table 3 compares its areal capacitance and cycling stability with previously reported Co_3_V_2_O_8_ and related vanadate materials. The P/S-CoVO electrode exhibits an areal capacitance of 13.714 F cm^−2^ at 8 mA cm^−2^, significantly higher than earlier reports, under comparable conditions. Furthermore, it maintains 83.68% of its initial capacitance over 12,000 cycles, demonstrating excellent long-term stability. These results indicate that the combination of PVP/SDS surfactant-assisted growth and direct deposition onto Ni foam effectively enhances charge storage and interfacial kinetics, outperforming conventional Co_3_V_2_O_8_ electrodes and related vanadate composites.

## 6. Electrochemical Performance of Asymmetric Supercapacitor Device

**Figure 10:**
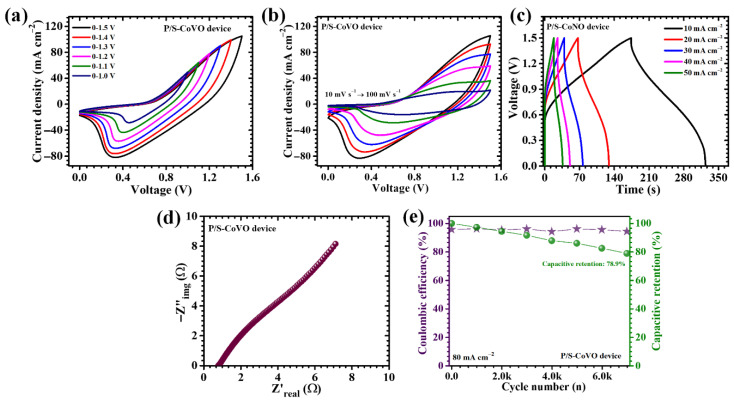
(**a**) CV curves of the P/S-CoVO//AC device recorded at different potential ranges from 0–1.0 V to 0–1.5 V, (**b**) CV curves of the P/S-CoVO//AC device recorded at scan rates of 10–100 mV s^−1^ within a potential window of 0 to 1.5 V, (**c**) GCD measurements at various current densities for the P/S-CoVO//AC device, (**d**) EIS analysis of the device, and (**e**) cyclic stability over 7000 GCD cycles for the P/S-CoVO//AC device.

To estimate the practical potential of the optimized P/S-CoVO electrode, an asymmetric pouch-type supercapacitor (APSC) was assembled using P/S-CoVO as the positive (faradaic) electrode and activated carbon (AC) as the negative (EDLC) electrode. Activated carbon was chosen for the negative electrode to provide a stable, high-surface-area double-layer counterbalance to the redox-active Co_3_V_2_O_8_, thereby creating a complementary hybrid energy-storage system. Both electrodes were deposited onto nickel-foam current collectors to ensure high electrical conductivity and mechanical robustness. The negative electrode slurry comprised activated carbon, acetylene black as a conductive additive, and PVDF binder dispersed in N-methyl-2-pyrrolidone (NMP). The slurry was homogenized, 2.0 mg cm^−2^ on an active area of 1 cm^2^, coated onto nickel foam, and dried under vacuum to remove residual solvent. The two electrodes were mass-balanced to approximate charge neutrality in the working voltage window, separated by porous filter paper saturated with 2 M KOH electrolyte, and hermetically sealed in a laminated pouch to prevent electrolyte loss and atmospheric contamination during cycling. Electrochemical characterization of the assembled device was carried out by CV, GCD, and EIS. To ensure an optimal operating voltage window, CV measurements were first conducted individually on the P/S-CoVO//AC device at different potential ranges from 0–1.0 V to 0–1.5 V, as illustrated in Figure 10a. As the potential window expanded, the CV profiles retained their quasi-rectangular shape without noticeable distortion or excessive current rise, confirming excellent electrochemical stability and the absence of parasitic side reactions such as electrolyte decomposition. Within the 0–1.5 V window, the device exhibited well-defined and reversible redox features, indicating that both the P/S-CoVO positive electrode and the AC negative electrode operated synergistically within safe electrochemical limits. Hence, 1.5 V was established as the optimized working potential for subsequent measurements, providing a balanced compromise between energy density enhancement and electrochemical safety. Further, CV scans collected over increasing scan rates (10 to 100 mV s^−1^, Figure 10b) exhibited quasi-rectangular profiles superimposed with broad redox features, consistent with the coexistence of capacitive behavior from AC and diffusion-controlled pseudocapacitance from P/S-CoVO. The device supported an extended aqueous operating window, enabling effective exploitation of both faradaic and non-faradaic storage mechanisms.

GCD curves (Figure 10c) showed the expected nonlinear charge–discharge curves characteristic of a hybrid device, with plateau-like regions reflecting redox activity of the P/S-CoVO positive electrode and near-linear regions corresponding to EDLC behavior of the AC negative electrode. Notably, the superior areal capacitance of the P/S-CoVO//AC device of 0.37 F cm^−2^ at 10 mA cm^−2^ with energy density of 0.115 mWh cm^−2^ (Table 4) translates a device-level enhancement in stored charge. EIS (Figure 10d) of the asymmetric device displayed a small semicircular region at high frequencies, denoting low interfacial charge-transfer resistance, followed by a steep, nearly vertical tail at low frequencies, which is indicative of a capacitive response and facile ion diffusion within the cell. The ESR was found to be only 0.78 Ω, confirming excellent electrical conductivity and rapid ion diffusion within the electrode structure. This low resistance is directly linked to the uniform porous network formed by the synergistic action of PVP and SDS, which collectively enhance both electron mobility and electrolyte accessibility. Durability testing under repeated GCD cycling at elevated current density of 80 mA cm^−2^ for 7000 successive cycles (Figure 10e) revealed excellent device stability. It retained a large fraction of its initial capacitance over numerous of cycles while maintaining high coulombic efficiency, demonstrating that the P/S-CoVO structure tolerates extensive ion insertion/extraction without significant mechanical or electrochemical degradation. The device demonstrated exceptional stability, retaining 78.9% of its initial capacitance and sustaining a high coulombic efficiency of 94.3% throughout the test. This excellent retention underscores the robust structural integrity and reversible redox activity of the hybrid electrode. The superior cycling stability can be attributed to the mechanically resilient and hierarchically porous nanoflower framework of the P/S-CoVO electrode, which effectively tolerates volumetric changes during repeated ion insertion and extraction. In summary, the P/S-CoVO//AC asymmetric pouch cell combines the high pseudocapacitance and ionic diffusivity of the hybrid Co_3_V_2_O_8_ electrode with the stable double-layer behavior of activated carbon to deliver a balanced device: extended voltage window, low internal resistance, efficient charge transfer, and robust long-term cycling. These results demonstrate the potential of dual-surfactant morphological engineering to bridge laboratory electrode optimization and practical, high-performance energy-storage devices.

## 7. Conclusions

This comprehensive study establishes a clear structure–property–performance relationship for CoVO electrodes modulated by surfactants, demonstrating how surfactant chemistry directs nanoscale morphology, charge transport, and electrochemical efficiency. Among all systems investigated, the dual P/S-CoVO electrode showed superior structural and electrochemical performance, owing to the combined effects of PVP’s surface-stabilizing capability and SDS’s pore-directing influence. This synergy produced a highly uniform, porous, and conductive nanoflower architecture, facilitating rapid ion diffusion and reversible redox reactions. Electrochemical characterization confirmed pseudocapacitive behavior dominated by diffusion-controlled kinetics with minimal resistance losses, as reflected by a low ESR of 0.52 Ω and enhanced diffusion coefficients. The P/S-CoVO electrode exhibited outstanding cycling stability, retaining ~83.7% of its initial capacitance after 12,000 cycles, highlighting mechanical robustness and interfacial stability. The assembled asymmetric device (P/S-CoVO//AC) further demonstrated practical relevance, delivering high energy and power densities and stable operation across an extended 1.5 V potential window. Overall, this study underscores that dual surfactant-assisted morphological engineering is an effective and scalable strategy for designing high-performance cobalt vanadate-based electrodes for next-generation supercapacitors.

## Figures and Tables

**Table 1:** Calculated diffusion coefficients, b-values, and series resistance of CoVO, P-CoVO, and P/S-CoVO electrodes.

Sample Code	Diffusion Coefficient (cm^2^/s) × 10^−8^		b-Value	ESR (R1) (Ω)	Rct (R2) (Ω)
	Oxidation	Reduction			
**CoVO**	7.02	4.72	0.56	0.802	12.89
**P-CoVO**	10.8	8.28	0.53	0.67	6.042
**P/S-CoVO**	20.46	14.05	0.51	0.52	2.134

**Table 2:** Comparison of areal capacitance, specific capacity, energy density, and power density of CoVO, P-CoVO, and P/S-CoVO electrodes.

Sample Code	I (mA cm^−2^)	Areal Capacitance C_A_ (F cm^−2^)	Capacity (mAh cm^−2^)	Energy Density ED (mWh cm^−2^)	Power Density PD (mW cm^−2^)
**CoVO**	8	5.172	0.251	0.088	2.37
	10	2.449	0.119	0.042	2.62
	20	2.416	0.117	0.041	5.07
	30	2.057	0.100	0.035	7.41
	40	1.763	0.086	0.030	9.31
**P-CoVO**	8	6.269	0.305	0.107	2.49
	10	3.298	0.160	0.056	2.89
	20	2.612	0.127	0.044	4.85
	30	2.449	0.119	0.042	7.14
	40	2.155	0.105	0.037	8.25
**P/S-CoVO**	8	13.714	0.667	0.233	2.61
	10	10.449	0.508	0.178	2.54
	20	7.837	0.381	0.133	5.20
	30	7.510	0.365	0.128	7.16
	40	5.224	0.254	0.089	8.89

**Table 3:** Comparison of performance metrics with previous literature.

Material	Areal Capacitance	Current	Stability	Ref.
Co_3_V_2_O_8_	3.76 F cm^−2^	1 mA cm^2^	-	[56]
Co_3_V_2_O_8_	3.5 F cm^−2^	1 mA cm^2^	87% retention (5000 cycles)	[57]
Ni, Bi, Co-Co_3_V_2_O_8_	285.65 F g^−1^	-	83.64% retention (5000 cycles)	[14]
Co_3_V_2_O_8_/CN_x_ composite	1.23 F cm^−2^	1 mA	87% retention (4000 cycles)	[34]
CO_3_V_2_O_8_	790 F g^−1^	1 A g^−1^	90.1% retention (10000 cycles)	[15]
Bismuth vanadate–V_2_O_5_	0.288 F cm^−2^	0.12 mA cm^−2^	99.7% retention (4000 cycles)	[58]
**P/S-CoVO**	**13.714 F cm^−2^**	**8 mA cm^−2^**	**83.68% retention** **(12,000 cycles)**	**This work**

**Table 4:** Areal capacitance, specific capacity, energy density, and power density of the P/S-CoVO asymmetric pouch-type supercapacitor device.

Sample Code	I (mA)	C_A_ (F cm^−2^)	C (mAh cm^−2^)	ED (mWh cm^−2^)	PD (mW cm^−2^)
**P/S-CoVO device**	10	0.369	0.077	0.115	1.37
	20	0.276	0.057	0.086	2.50
	30	0.197	0.041	0.062	2.92
	40	0.142	0.030	0.044	3.20
	50	0.102	0.021	0.032	3.19

## Data Availability

The data presented in this study are available on request from the corresponding author due to privacy reasons.

## Supplementary Materials

The following supporting information can be downloaded at https://www.mdpi.com/article/10.3390/mi16121334/s1, Figure S1. (a1–a3) FESEM micrographs of the SDS-assisted CoVO (SDS–CoVO) nanostructures at different magnifications.
