# Supplementary material for: Dual Surfactant-Assisted Hydrothermal Engineering of Co3V2O8 Nanostructures for High-Performance Asymmetric Supercapacitors
Source: Micromachines (Basel). 2025 Nov 27;16(12):1334. doi: 10.3390/mi16121334 (PMC12734452; doi:10.3390/mi16121334)
Supplement: Supplementary file 1 [file micromachines-16-01334-s001.zip › micromachines-4016030-supplementary.pdf]

Dual Surfactant-Assisted Hydrothermal Engineering of $Co_3V_2O_8$ Nanostructures for High-Performance Asymmetric Supercapacitors

Pritam J. Morankar [a], Aditya A. Patil [a], Aviraj Teli [b], Chan-Wook Jeon [a*]

a School of Chemical Engineering, Yeungnam University, 280 Daehak-Ro, Gyeongsan 38541, Republic of Korea
b Division of Electronics and Electrical Engineering, Dongguk University-Seoul, Seoul, 04620, Republic of Korea
* Corresponding Authors Email: cwjeon@ynu.ac.kr

Supplementary information

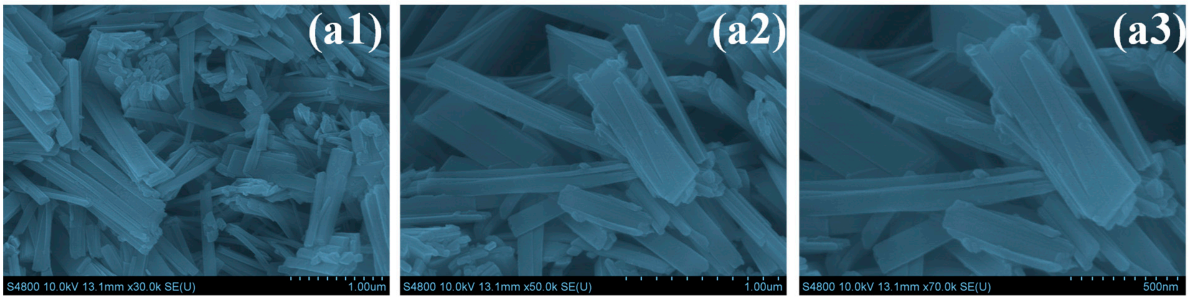

Figure S1: (**a1**–**a3**) FESEM micrographs of the SDS-assisted CoVO (SDS–CoVO) nanostructures at different magnifications.
